# A Review Study of the Participation of Late Domains in Sorting and Transport of Viral Factors to Exosomes

**DOI:** 10.3390/life13091842

**Published:** 2023-08-31

**Authors:** Manuel Adrián Velázquez-Cervantes, Yazmín Rocío Benítez-Zeferino, Arturo Flores-Pliego, Addy Cecilia Helguera-Repetto, David Eduardo Meza-Sánchez, José Luis Maravillas-Montero, Guadalupe León-Reyes, Javier Mancilla-Ramírez, Jorge Francisco Cerna-Cortés, María Isabel Baeza-Ramírez, Moises León-Juaárez

**Affiliations:** 1 Laboratorio de Virología Perinatal y Diseño Molecular de Antígenos y Biomarcadores, Departamento de Inmunobioquímica, Instituto Nacional de Perinatología, Mexico City 11000, Mexico; manugenes18@hotmail.com (M.A.V.-C.); bezyqcb@hotmail.com (Y.R.B.-Z.); 2 Laboratorio de Biomembranas, Departamento de Bioquimica, Escueala Nacional de Ciencias Biológicas, Instituto Politécnico Nacional, Mexico City 11340, Mexico; isabelbaeza@yahoo.com; 3 Laboratorio de Microbiología Molecular, Departamento de Microbiología, Escuela Nacional de Ciencias Biologícas, Instituto Politécnico Nacional, Mexico City 11340, Mexico; jorgecerna1008@gmail.com; 4 Departamento de Inmunobioquimica, Instituto Nacional de Perinatología, Mexico City 11000, Mexico; arturo_fpliego@yahoo.com.mx (A.F.-P.); ceciliahelguera@yahoo.com.mx (A.C.H.-R.); 5 Red de Apoyo a la Investigación, Coordinación de la Investigación Científica, Universidad Nacional Autonóma de México, e Instituto Nacional de Ciencias Médicas y Nutrición Salvador Zubirán, Mexico City 04510, Mexico; dmeza@cic.unam.mx (D.E.M.-S.); maravillas@cic.unam.mx (J.L.M.-M.); 6 Laboratorio de Nutrigenómica y Nutrigenética, Instituto Nacional de Medicina Genómica (INMEGEN), Ciudad de México 14610, Mexico; greyes@inmegen.gob.mx; 7 Escuela Superior de Medicina, Instituto Politécnico Nacional, Mexico City 113440, Mexico; drmancilla@gmail.com; 8 Hospital de la Mujer, Secretaría de Salud, Mexico City 11340, Mexico

**Keywords:** exosomes, viral infection, late domain, biogenesis

## Abstract

Cellular communication depends heavily on the participation of vesicular systems generated by most cells of an organism. Exosomes play central roles in this process. Today, these vesicles have been characterized, and it has been determined that the cargo they transport is not within a random system. In fact, it depends on various molecular signals and the recruitment of proteins that participate in the biogenesis of exosomes. It has also been shown that multiple viruses can recruit these vesicles to transport viral factors such as genomes or proteins. It has been shown that the late domains present in viral proteins are critical for the exosomal selection and biogenesis systems to recognize these viral proteins and introduce them into the exosomes. In this review, the researchers discuss the evidence related to the characterization of these late domains and their role in exosome recruitment during viral infection.

## 1. Introduction

Eukaryotic cells are complex structures formed by an organization of intracellular organelles. This complexity can increase if these cells also have extracellular organelles that are released into their microenvironment. Together, these membranous structures released by cells are called extracellular vesicles (EVs), which are produced under normal physiological conditions or during infectious or pathological processes [1]. EVs can be classified into ectosomes and exosomes. Ectosomes are vesicles that emerge from the surface of the plasma membrane through outward budding; here, we can include microvesicles, microparticles, and long vesicles that range in diameter from 100 to 500 nm [2,3]. Exosomes are EVs with an average size of 100 nm, ranging from 40 to 160 nm, and are characterized by an endosomal origin. A particular proprietary feature of exosomes is their interconnection with other vesicles, such as multivesicular vesicles or different organelles, which may contribute to the diversity of molecules and signaling exosomes [4,5].

Throughout history, there have been references made to vesicles. However, the first mention of these vesicles was in 1960: the characterization of secreted small vesicles, with a size of 100 nm, which originated from chondrocytes by budding directly from plasma membranes during the formation of hydroxyapatite crystals [6,7]. Studies on other cell types have shown the relevant functions of EVs in several roles such as coagulation, the calcification of bones, and the repair of pathological conditions such as arthritis and autoimmune disorders [5,8,9,10,11]. A pioneering study by Trams et al. revealed the presence of vesicles of different sizes secreted by cells and their potential physiological roles. The characterization of two of these diversely sized vesicles by differential centrifugation revealed the presence of two separate vesicular entities. Over time, these methods have allowed the identification of proteins, nucleic acids, lipids, and glycoproteins that enrich these vesicular structures [12].

Although cells have a secretome that is well characterized by several elements, exosomes are identified today as one of the vesicular mediators with broad biological functions. They are involved in intracellular communication at the local and systematic levels via the transfer of functional proteins, metabolites, and nucleic acids to recipient cells. In addition, exosomes influence various biological processes, such as embryogenesis, immune response, tissue repair, antigen presentation, programmed cell death, angiogenesis, inflammation, coagulation, and pathological processes in cardiovascular diseases, infectious diseases, neurodegeneration, and cancer [13,14,15,16,17,18].

## 2. Biogenesis of Exosomes

**Figure 1:**
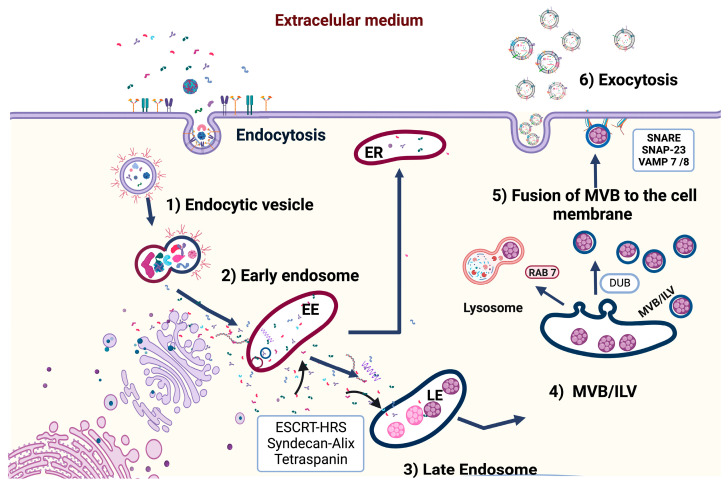
Biogenesis of exosomes. Fusion of primary endocytic vesicles is the first step of early endosome (EE) formation EEs: returning to the plasma membrane or changing into late endosomes (LE) MVBs. Protein sorting of ILVs can be ESCRT-complex-dependent or ESCRT-independent mechanisms. Later, targeted ILVs are prepared to be degraded within a lysosome or rescued by DUBs. Rab27A and Rab27B are crucial mediators to lead the MVBs toward the cell periphery. Finally, the SNARE complex helps the fusion of MVBs with the plasma membrane to release ILVs into the extracellular space, which are now called exosomes.

The origin of these vesicular structures requires two well-defined events. The first occurs in the plasma membrane during the endocytosis process. Subsequently, during endocytic trafficking in the early endosome, these vesicles, obtained from the plasma membrane, provide the first classification and fate of cargo molecules. Three routes have been defined for cargo molecules in early endosomes. First, the molecules that require recycling are targeted for movement to the tubular peripheral domains of endosomes. These vesicles can finally merge with the Golgi apparatus network or move toward the plasma membrane via endosomal recycling. When cargo is not directed toward recycling, these molecules are concentrated in the early endosome vacuolar regions. Their fate is related to the endosomal maturation pathway until they reach the late endosomes. Two routes are taken: fusion with the lysosome and subsequent degradation or fusion with the plasma membrane and exosome release (Figure 1) [18,19,20,21]. During vesicular trafficking, endosomal membranes undergo a series of changes and modifications that allow their mobility and classification via the endosomal route. The membrane of the early endosome is enriched in phosphatidyl inositol 3-phosphate (PI3P), as well as phosphatidyl inositol 4-5 bisphosphate (PI(4,5)P2). There is also an exchange of sphingomyelin with ceramides and a change in proteins, such as RAB5 to RAB11, which regulate traffic toward the late endosome. Continuing with these changes during endosomal maturation, certain areas of the endosomal membrane begin to invaginate and bud in the direction of the cytoplasm toward the intraluminal space of the endosome. At this point, the intraluminal vesicles were generated. In this section, the cargo is enclosed; these structures appear as multivesicular bodies or body formation proteins (MVBs) in the late endosome. Additionally, the classification of proteins occurs via two different processes: the endosomal sorting complexes required for transport (ESCRT) complex or a mechanism independent of this system (ESCRT-independent mechanisms) [5,18,22,23]. When the MVBs follow a pathway leading to fusion with the lysosome, the cargo within the intraluminal vesicles (ILVs) will begin a degradation process. However, if MVBs follow a path to the plasma membrane, ILVs are secreted from the cell and are considered exosomes [18,23].

## 3. ESCRT Factor-Dependent and Independent Pathways in Exosomal Biogenesis

ESCRT multiprotein complexes are highly conserved and consist of class E vacuolar protein sorting (VPS) proteins, which assemble into four distinct complexes: ESCRT-0, I, II, and III. They are associated with each other, as well as with other accessory proteins such as vesicle trafficking 1 (VTA1) and apoptosis-linked gene 2 (ALG-2) interacting protein X (ALIX). Interestingly, this protein machinery coordinates molecular binding and membrane deformation events that result in the biogenesis and cargo recruitment of ILVs [4,24,25,26]. The components of each ESCRT complex have been characterized and are listed below. The ESCRT-0 complex is made up of hepatocyte growth factor-regulated tyrosine kinase substrate (HRS), which recognizes monoubiquitinylated cargo proteins and interacts in a complex with signal-transducing adapter molecules (STAM). ESCRT-I consists of tumor susceptibility 101 (TSG101), VPS28, VPS37, and multivesicular body subunit 12 (MVB12). ESCRT-II consists of EAP30, EAP20, and ESP45; and ESCRT-III consists of CHMP6, CHMP4, CHMP2, and CHMP3 [27,28].

**Figure 2:**
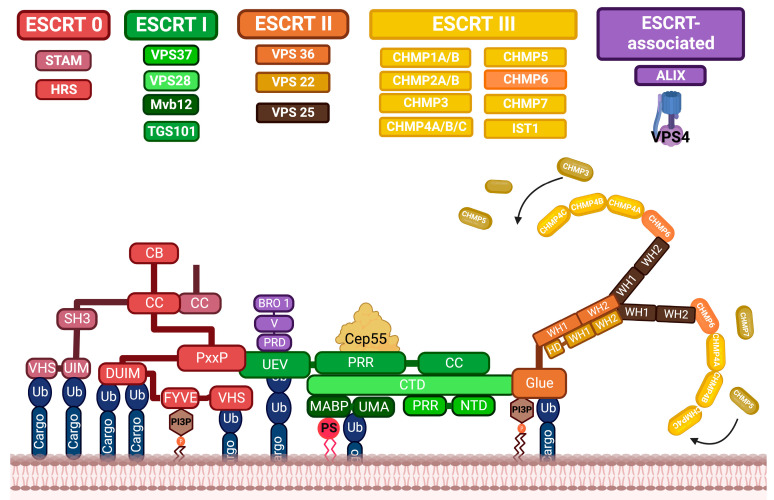
Protein associations of the ESCRT complex. ESCRT complexes are recruited sequentially into the endosome and recognize ubiquitinated transmembrane proteins, shifting the load from one complex to the next to facilitate sorting to the MVB vesicles. ESCRT-0 binds to PI3P and groups ubiquitin membrane proteins across ubiquitin-binding domains (VHS, UIM, and DUIM). While ESCRT-I is recruited by ESCRT-0. PxxP within the carboxyl end of HRS is associated with an E2 variant domain of ubiquitin (UEV) in TSG101. ESCRT-II interacts through the VPS36 Glue domain with ESCRT-I, PI3P, and charge. The VP25 subunit of ESCRT-II serves as a nucleation point for the staggered assembly of the ESCRT-III filament complex, which sequesters the load and drives the inward budding of the vesicle. Clathrin Binding (CB); Coiled-Coil (CC); Double-Sided Ubiquitin-Interacting Motif (DUIM); Phosphatidylinositol-3-Phosphate (PI3P); Src homology-3 (SH3); Ubiquitin Interacting Motif (UIM).

The cellular process that initiates the ESCRT-dependent pathway is determined by the high abundance of PI3P in the early endosomal membrane, which allows the recruitment and binding of HSR in ESCRT-0 via a motif known as FYVE. Subsequently, the ESCRT-0 subunits HRS and STAMs interact directly with each other and begin to identify and join the ubiquitinated cargo to be sequestered. Additionally, HRS harbors a PSAP motif that facilitates the recruitment of ESCRT-0 to ESCRT-1 via TSG101. ESCRT-I VSP8 then binds with ESCRT-II EAP45; in this part, the ESCRT-I MVB12 protein also regulates the selection of mono- and di-ubiquitinylated charges within ILVs. The final step involves the assembly of ESCRT III, which begins when ESCRT-II binds to ESCRT III CHMP6 and participates in the polymerization and activation of ESCRT-III via CHMP4, and the subsequent recruitment of CHMP2 and CHMP3 units into the cell endosomal membrane. Within the ESCRT-III complex, CHMP4 plays an essential role in membrane deformation, leading to internal budding and generation of ILVs [18,28,29]. In addition, it has been described that CHMP4 can polymerize, generating spiral filamentous structures that mediate the production of negative curvatures in the membrane. Once membrane fusion occurs, the ESCRT-III complex disassembles when its polymers translocate through the central pore of VPS4 ATPase. However, the role of VPS4 is not unique to the above process because it also regulates membrane remodeling processes to stabilize regions in growing ILVs. Other cellular factors participate in the formation of ILVs and are involved in ESCRT-dependent pathways. ALIX is one of the best-characterized proteins in this process; it is involved in mediating the ESCRT-III interaction independently without requiring the assembly of upstream ESCRT complexes, thus classifying and delivering tetraspanins to exosomes by ALIX. Additionally, ALIX interacts with syndecans, a transmembrane protein that serves as a scaffold for synteins and participates in the membrane budding steps of ILV biogenesis (Figure 2) [18,30].

In eukaryotic cells, the generation of MVBs may have an alternative mechanism without the participation of the ESCRT complex, where the involvement of some proteins and lipids has been identified. Perhaps a different characteristic of this alternate pathway is the larger size and smaller number of encapsulated ILVs that are irregular in shape and size. Among the cellular factors that may be associated with this independent pathway, tetraspanins have been shown to participate in various events of exosome biogenesis because they direct cargo to MVBs by compartmentalizing the endosomal membrane via domains enriched by tetraspanins. The tetraspanin CD63 is most frequently identified in vesicular transport. Furthermore, among the lipids that participate in this pathway, ceramides and sphingolipids participate in membrane deformation. This step is organized in the plasma membrane within regions enriched with sphingolipids, cholesterol, and proteins, spontaneously generating negative curvatures in the membrane, leading to the generation of IVLs without the participation of ESCRT-III [30]. Some of the cellular factors that have been characterized as participating in this pathway of IVL biogenesis are GTPase RAB31 and ceramide transfer protein, which play a role in triggering the budding of membranes in these lipid regions and the transfer of these types of lipids in the membranes of the endocytic pathway from the Golgi apparatus and endoplasmic reticulum [23,31].

## 4. Exosome Composition

**Figure 3:**
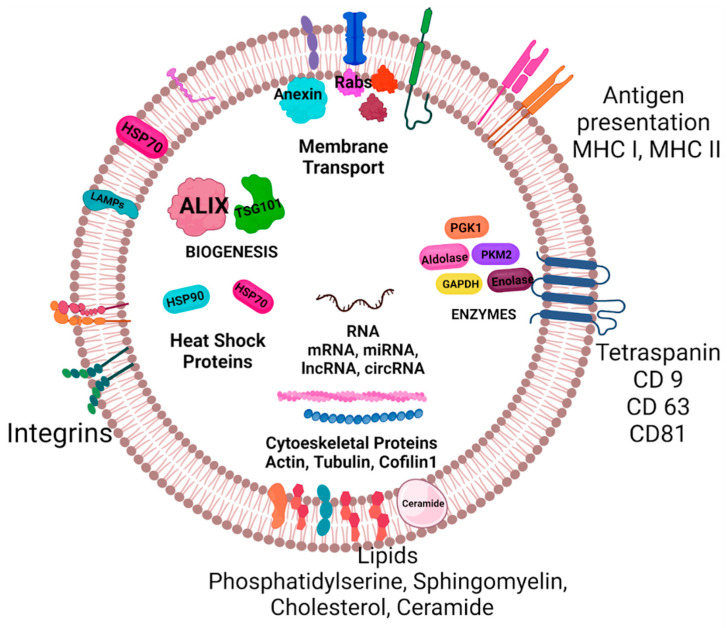
Molecular Composition of exosomes. Exosomes are rich in a wide variety of proteins (41,860), lipids (1116), and nucleic acids (3408 mRNAs and 2838 miRNAs). The main membrane-bound, and cytosolic proteins incorporated in exosomes are members of the tetraspanin family (CD9, CD63, and CD81), an endosomal sorting complex required for transport (ESCRT, Alix, TSG101), integrins, heat shock proteins (Hsp), actin and flotillins. Their unique composition depends on the type of cell and physiological or pathological state.

Various proteins, lipids, and nucleic acids enrich the biochemical composition of exosomes. In addition, their composition is determined by factors such as cell type, physiological conditions, or the pathological state of the cell system from which they are isolated. In this review, the platform known as the exosome database described 9769 proteins, 3408 mRNAs, 2838 miRNAs, and 1116 lipids that have been reported as biochemical elements that make up exosomes [32]. Different types of RNAs have been characterized in exosomes, for example, mitochondrial RNAs, long non-coding RNAs (IncRNAs), and their fragments: miRNAs, small nuclear RNAs, small nucleolar RNA, and ribonucleoproteins (hnRNP), such as A2B1 and HNRNPA2B1. All of these could function as regulators of gene expression in the receptor cells of these vesicles. However, they are generally composed of a phospholipid membrane containing tetraspanins, thermal shock proteins (HSP), GTPases, Flotilin, adhesion molecules, components of the ESCRT complex, MVB proteins responsible for membrane transport and fusion, metabolic enzymes, phosphatidylcholine, phosphatidylserine, phosphatidylethanolamine, sphingomyelin, ceramides, diacylglycerides, and lipids-rafts (Figure 3). It is speculated that the amount and location of cholesterol components may influence the fate of these [18,33,34].

Tetraspanins are required for cell penetration, invasion, and fusion. They do not possess their own catalytic activities but rather facilitate the trafficking, function, stability, and oligomerization of other membrane proteins. Exosomes are rich in CD81, CD82, CD37, and CD63 and are frequently used as exosomal markers, along with other exosomal tetraspanins, such as CD9. CD63 is a protein that circulates between the plasma membrane and endosomal compartments and plays a role in the classification of loads [35,36]. CD9 and CD81 are expressed in a wide variety of cells and are considered “molecular facilitators” that interact with specific proteins involved in the development, proliferation, activation, and motility of somatic cells. As for CD9, it consists of four transmembrane domains, including one intracellular end and two extracellular loops. It performs a wide variety of biological activities, such as cell adhesion, motility, metastasis, growth, signal transduction, hematopoietic stem cell differentiation, and sperm and egg fusion [37].

For the components of the ESCRT complex, we have TSG101 and ALIX. TSG101 is a multidomain protein encoded by tumor susceptibility gene 101 that participates in various intracellular processes, such as transcription regulation, cell proliferation and division, ubiquitination and intracellular movement of proteins, normal tissue homeostasis, and tumorigenesis. TSG101 is mostly found in the cytoplasm, regardless of the cell cycle stage; however, during the late S phase, a fraction of the protein is found in the nucleus and colocalizes with the mitotic spindle during cell division. ALIX and AIP1 are cytosolic proteins that participate in the regulation of various cellular mechanisms such as the classification of proteins in endosomes, cell adhesion, and apoptosis [29,38].

## 5. Virus Modifications of Exosomes

Viral infections can carry viral or cellular factors that favor pathogenicity, dissemination, replication, and antiviral effects that can trigger several pathogenic mechanisms. In this sense, it has been observed that the tick-borne flaviviruses Langat Virus (LGTV) and West Nile Virus (WNV) hijack the exosomal pathway of arthropods to transport viral factors. Exosomes from LGTV-infected tick cells were used to incubate human HaCaT epithelial cells, finding that there is an active infection as viral RNA and envelope protein are transported, while West Nile was found able to be transported in arthropod exosomes WNV RNA, and thus these viral factors transported by arthropod exosomes have an infective capacity in murine and human cell lines [39]. Other flaviviruses that have the ability to transport viral factors through exosomes are Dengue Virus (DENV-2) and Zika Virus (ZIKV). Since it was found that within the exosomes produced in A549 cells, the NS1 protein was transported, this fact opens the possibility that this protein travels through the exosomes to reach the endothelial barriers and damage the cellular environment, which is the main target of these flaviviruses [40]. In the case of the Hepatitis C virus (HCV), there is evidence that viral RNA, core protein, and E2 are transported within the exosomes of Huh7.5.1 liver cells. These viral factors caused a productive infection, and when the infection was treated with neutralizing antibodies, it was not affected due to the transport of HCV viral factors by the exosomes, evading the immune response [41]. One of the most studied viruses with sequestration and transport of viral factors is Human Immunodeficiency Virus (HIV-1). It has been described that the infection of monocyte-derived macrophages produces exosomes containing the HIV-1 viral particle and the Gag protein, and that these exosomes have infective activity in other cell lines such as Hela cells [42]. Also with HIV-1, it was found that the Nef protein of this virus is within the exosomes produced by the infection, and it was observed that nearby cells were more vulnerable. Therefore, the exosomes carrying Nef were able to promote death in CD4 T lymphocytes [43]. In the C6/36 cells infected with ZIKV, exosomes containing viral RNA and the envelope protein of this virus are produced. These viral factors evade the immune response and thus easily reach HMEC-1 endothelial cells and THP-1 monocytes. Likewise, the regulation of proinflammatory proteins such as tissue factor (TF), protease-activated receptor-1 (PAR-1), ICAM-1, and TNF-α are involved in increased hyperpermeability and endothelial damage [44].

**Figure 4:**
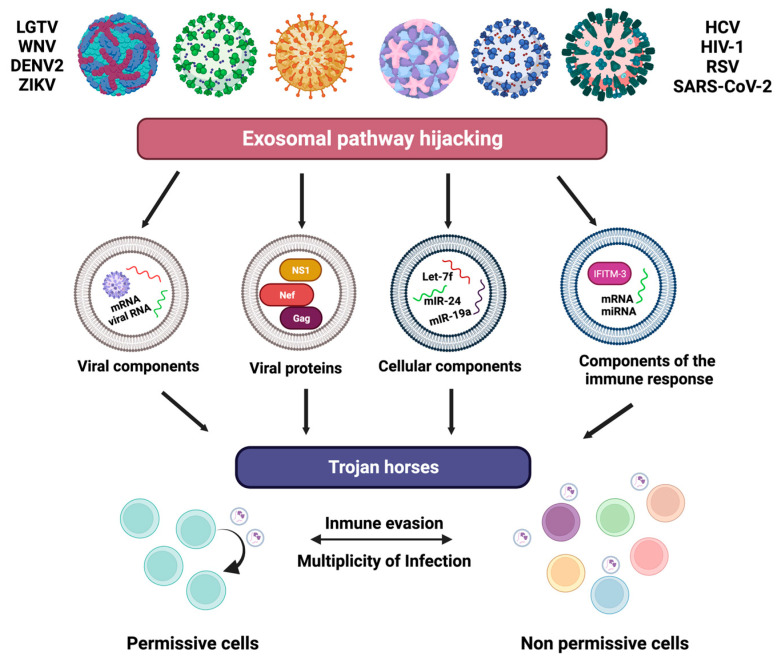
Hijacking of the exosomal pathway by viruses. The association between exosomes and viruses is not clear; however, it has been observed that exosomes before a viral infection can act by facilitating or inhibiting the infection. In the first case, it improves infectivity of many viruses, such as LGTV, WNV, DENV-2, ZIKV, HCV, HIV-1, RSV, and SARS-CoV-2, by hijacking the exosomal pathway to transport viral, cellular or immune response factors to other neighboring or distant cells, exosomes being a Trojan horse that allows the dissemination and replication of viruses without the detection of the immune system, and alter non-permissive cells for better propagation of these viral agents. In the second case, exosomes can act as antigen-presenting vesicles and activate CD4 T cells.

Viral proteins are not the only factors that are sorted within exosomes during infections by these pathogens. These factors aid in their dissemination, replication, or pathogenicity in the organism, but they are not the only ones that are as described below exosomes in a virus-infection environment and can have the duality of carrying cellular factors that aid in antiviral or proviral response. It has been described that the Respiratory Syncytial Virus (RSV), in a model of A549 cells, can have exosomes isolated from this infection. Chemokines such as MCP-1, IP-10, and RANTES, were carried in these exosomes, which suggests the activation of the innate immune response and antiviral effect of the isolated exosomes. Thus, exosomes isolated from RSV infection in A549 cells have the potential to activate the innate immune response by activating cytokines and chemokines from human PBMC monocytes, whereas an extrapolation of this same model of exosomes isolated from A549 RSV-infected, but the primary culture of epithelial alveolar respiratory tract cells, found that the isolated exosomes contained the same miRNA profile as Let-7f, -7i, miR-24, -31, and -221 that are part of the regulation of replication of RSV and porcine reproductive and respiratory syndrome virus infection (PPRSV). However, in primary culture, the levels of these miRNAs were increased, so this approach to exosomes during RSV infection allows us to assess the possible potential of exosomes to regulate the pathogenesis and immune response of RSV [45]. The isolation of exosomes infected with DENV-2 from various cell lineages such as HUVEC and HepG2, allowed us to visualize that the function of these exosomes is to transport antiviral factors such as IFITM 3, which has effects to suppress the entry of various enveloped viruses. Therefore, this study allowed us to relate that the exosomes produced by DENV-infected cells carry antiviral factors to other cells that are not infected [46]. It was recently shown that exosomes from the plasma of patients with mild or severe COVID-19 have the ability to induce NLRP3, caspase-1, and IL-1β mRNA expression in microvascular epithelial cells (HMEC-1) and hepatic endothelial cells (TMNK-1), resulting in the activation of Casp1 and subsequently IL-1β through the NLRP3 inflammasome in these endothelial cells. Thus, plasma exosomes from COVID-19 patients would have the ability to regulate SARS-CoV-2 virus-induced disease that develops venous thromboembolism, coagulopathy in epithelial cells, multiple organ damage, and induce strong molecular signals in distant cells for immunopathogenesis. [47]. While exosomes produced during HCV infection of immortalized human hepatocytes (IHH) showed content that can carry profibrogenic factors that trigger end-stage liver disease, it was evidenced that miR-19a complies with this profibrogenic regulation. This miRNA was found within the exosomes of IHH cells to be captured by another cell lineage like human hepatic stellate cells LX2 and, upon entering the miR-19a via exosomes, caused in LX2 cells the activation of SOCS-activating TGF-β through the STAT3 pathway, which is involved in the increase in liver lesion so that the delivery of exosomes produced by HCV is important for the development of liver fibrosis damage [48]. It was observed that exosomes maintain cellular homeostasis by transporting antiviral factors that combat infection by various viruses. Interestingly, viruses can recruit exosomal biogenesis machinery for viral or cellular elements to distribute into these vesicles, and with them be a Trojan horse to transport these factors and aid in their dissemination and pathogenicity in permissive and otherwise differently located cells in the organism (Figure 4). In the next section, the focus is on describing the evidence associated with the late domains present in viral proteins and their role in the recruitment of the exosomal machinery.

## 6. Sorting of Cell Factors in Exosomes

**Figure 5:**
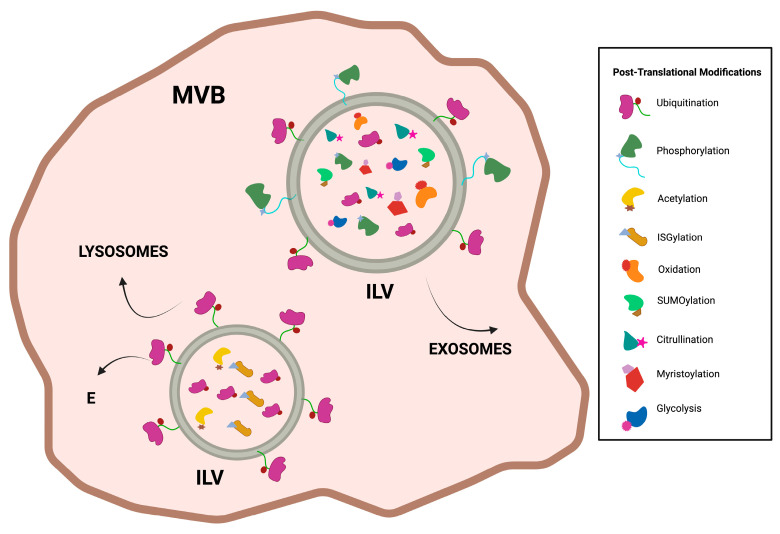
Schematization of sorting by post-translational modifications in exosomes. It shows which membrane proteins can assist ubiquitination and phosphorylation for incorporation into exosomes. It has been found that ubiquitination of HLA-G, H1.2, H1.3, Esrin, and HSP70 helps their sorting into exosomes, other posttranslational modifications that lead to this same process are phosphorylation of ARF6, SUMOylation of α-synuclein, oxidation of γ-synuclein, citrullination of α-fibrin, and fibrinogen and myristoylation of Tya. While ubiquitination of MCH-II regulates lLVs to take the lysosomal degradative pathway, other posttranslational modifications regulating this degradative pathway are acetylation of GRP78 and ISGylation of TSG101.

Cellular proteins can be transported by exosomes, according to the cellular context, to maintain homeostasis for pro-inflammatory and anti-inflammatory processes [21]. These sorting events occur based on the post-translational modifications of proteins, such as ubiquitination, SUMOylation, ISGylation, phosphorylation, oxidation, acetylation, citrullination, myristylation, and glycolysis (Figure 5) [21,49]. This is due to the important involvement of the ESCRT complex, which sorts ubiquitin-tagged proteins and their transport to ILVs and subsequent travel in exosomes [50]. This process begins when ESCRT-0, which contains a ubiquitin-binding domain (UBD) in its HRS and STAM 1/2 subunits through its FYVE domain recognizes ubiquitin-tagged proteins [51]. HRS also contains a PSAP domain that interacts with TSG101 of ESCRT-I and carries ubiquitin-tagged proteins within ILVs [52]. Once these sorting processes begin simultaneously, ESCRT-I recruits ESCRT-II to activate and associate with ESCRT-III; the latter is coupled by TSG101 and ALIX, which serves as an intermediate between ESCRT I and III as it associates with TSG101 of ESCRT-I and CHMP4A of ESCRT-III [53,54,55]. Subsequently, proteins must be deubiquitinated to remain in ILVs; to uncouple this signal, ESCRT-III is critical, as it recruits proteins that uncouple ubiquitin [45]. In this context, exosomes isolated from myeloid suppressor cells were enriched with ubiquitinated proteins, such as histones H1.2, H1.3, HSP70, and Ezrin [56]. Another ubiquitin-containing protein to be classified within exosomes is the non-classical human leukocyte antigen G (HLA-G); exosomes isolated from ascites and pleural exudates of patients with aplastic anemia HLA-G-positive showed that this protein was enriched by ubiquitin [57].

In contrast, ubiquitination is not the only pathway to reach exosomes, as ubiquitinated proteins can evade deubiquitination and thereby reach the lysosomal pathway. Ubiquitination in dendritic cells leads to endocytosis and MHC-II sorting to ILVs for removal by lysosomes, thus opening the possibility that another form of protein sorting exists in exosomes [58]. One alternative evidence for ubiquitination is phosphorylation, as ARF6 transport was observed in tumor cells, and this modification regulates exosome loading [59]. SUMOylation, which utilizes a small ubiquitin-like modifier, also shows the property of sorting proteins into exosomes, as it was found that when this modification is present in α-synuclein, it can be targeted and sorted into exosomes, within the context of Parkinson’s disease [60]. ISGylation is another post-translational modification similar to ubiquitin that allows the release of exosomes, as it regulates the number and secretion of exosomes. It was observed that ISGylation of TSG101 induces their aggregation and degradation and thereby modifies exosome production, so its function is believed to be the control of exosomes [61]. The oxidized-synuclein protein is secreted by exosomes produced by neuronal cells, which can be targeted for glial cells and cause intracellular protein aggregation, indicating that oxidation is important for the transport within exosomes [62]. GRP78 expression promotes the phenotypic features of cancer, and this protein is secreted by exosomes from cancer cells by inhibiting histone deacetylase inhibitors, blocking the release of GRP78-rich exosomes and their aggregation in the endoplasmic reticulum (ER), which promotes sorting in the exosomes [63]. Citrullination is a post-translational modification of peptidyl-arginine to peptidyl-citrulline, and in synovial fluid exosomes of fibrin and fibrinogen, were found to play an important role in the transport of these proteins in exosomes to regulate rheumatoid arthritis [64]. Myristoylation, which is based on the binding of a myristoyl molecule, binds covalently to an amino group of glycine and is also involved in the sorting of exosomes into the Tya protein, where this post-translational modification is targeted and transported in exosomes in vitro [65]. In addition to these post-translational modifications, glycosylation occurs because the galectin-3 binding protein of sialoglycoprotein (LGALS3BP) is found within exosomes that are abundant in glucans and these LGALS3BP sites. Therefore, it is possible for other proteins that are glycosylated to be transported in these exosomal vesicles [66]. However, this type of sorting is related to viruses, as it has been described that some viruses could take this strategy to carry viral factors inside exosomes. It has also been observed that some ubiquitins are important for the quasi-envelope and release viral, such is the case of ITCH, a member of the NEDD4 HECT domain E3 ubiquitin ligase family, which is associated with the pX c terminal sequence of the Hepatitis A (HAV), VP1 capsid of protein independently of ALIX. This interaction allows the quasi-envelope and release of eHAV (quasi-envelopment hepatovirus) from infected cells [67]. Studies in HCV show that the NS2 protein is ubiquitinated by E3 ubiquitin ligase and this posttranslational modification allows interaction with HRS and possibly allows entry of HVC into exosomes [68]. In Herpes Simplex Virus 1 (HSV-1), it is shown that the glycoprotein gB of this virus possesses ubiquitination that causes the HLA-DR antigen to become HLA-DR antigen and binds to this protein, and this gB-DR complex enters the exosome using gB ubiquitination, causing an alteration in the presentation of antigen by the MHC-II processing pathway and to evidence the immune system [69]. The Epstein–Barr virus (EBV) LAMP-1 protein has been found to play a role in sorting this virus into exosomes, as it associates with CD63, a tetraspanin involved in exosome biogenesis, but cellular LAMP-1 has been described to ubiquitinate to load into exosomes, so it is possible EBV LAMP-1 may have this ubiquitination ability to bind to CD63 of exosomes [70,71].

This type of sorting opens a panorama for further study of the behavior and dynamics of cellular and viral protein sorting and provides a broader picture of the functions of exosomes in the transport of cellular or other factors, such as viruses.

## 7. Late Domains

**Figure 6:**
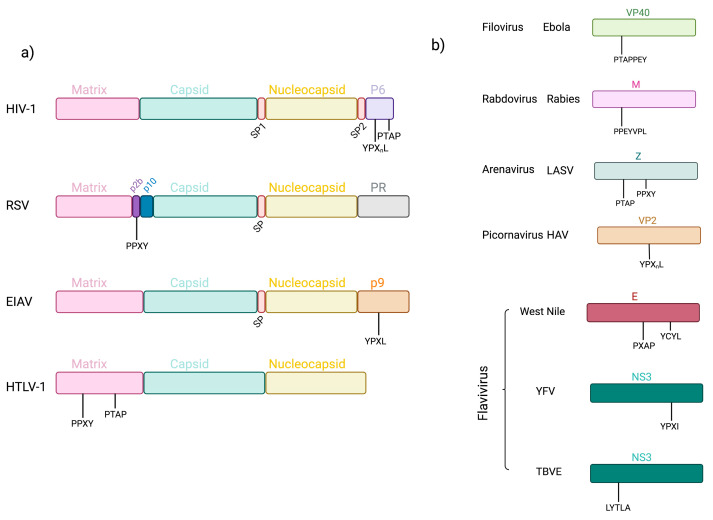
Schematization of the Gag protein and Late domains in other viruses. (**a**) The arrangement of the Gag protein of some retroviruses is shown, the matrix, cápside, and nucleocapsid proteins are conserved, and the location of the late PT/SAP, PPXY, and YXXL/YPX_n_L domains is shown. (**b**) Diversity of viral proteins other than Gag show late domains. There are different groups of viruses such as filoviruses, rhabdoviruses, arenaviruses, picornaviruses, and flaviviruses that show similarity in budding functions as in retroviruses.

One of the unknowns of how viral factors are recruited by the biogenesis machinery of exosomes is still not entirely clear. Cellular proteins use various post-translational modifications, or in the case of RNA, there are certain motifs for their sorting [21]. Regarding viral proteins, there is evidence that certain domains present in these proteins are essential for interaction with proteins involved in the formation of exosomes. Late domains were first identified in the Gag polyprotein (comprising mainly matrix, capsid, and nucleocapsid domains) of some retroviruses (Figure 6a), and later in arenaviruses, picornaviruses, rhabdoviruses, filoviruses, and flaviviruses (Figure 6b). The name of these late domains was assigned because these sequences played a final role in the budding process of the aforementioned viruses. These domains are PT/SAP, PPXY, and YXXL/YPX_n_L, where X can be any amino acid and _n_ can vary in length [72,73,74]. In this sense, it was observed that in the Gag protein of HIV-1, the p6 region shows two late domains, PTAP and YPX_n_L, and these are associated with proteins involved in the biogenesis of exosomes as reported with PTAP that interacts with the TSG101 component of the ESCRT machinery and helps the exit of this virus from the cell [75]. While the YPX_n_L domain, in the p6 region of the Gag protein, binds to ALIX, which in turn interacts with TSG101, it has been observed that in the absence of the PTAP domain, YPX_n_L and ALIX facilitate the release of HIV-1 because they interact with the ESCRT-III complex, which is recruited in the twinning regions together with VPS4 ATPase. This leads to the rearrangement of the membrane and its fission and drives the disassembly of ESCRT-III, thereby releasing viral particles [74,76]. In RSV, it was found that in the Gag protein, the p2b peptide was associated with virus release and, consequently, this function was regulated by a PPXY domain, which interacts with the E3 ubiquitin ligase Nedd4. This association is similar to that seen with the YPX_n_L domain of HIV-1, where Nedd4-1 was observed to interact with ALIX and assist in viral particle release, which is similar to RSV and the interaction of PPXY and Nedd4 [77,78].

In the equine infectious anemia virus (EIAV), there also is a Gag protein being a retrovirus. It was found that the presence of the YPXL domain was in the p9 region of its Gag protein and that this region interacts with ALIX. This interaction fulfills the same functions observed in p6 of HIV [79,80]. Another retrovirus studied was the human T-cell leukemia virus type 1 (HTLV-1) where it was found that the M region of the Gag polyprotein shows two late domains, PPXY and PTAP. As demonstrated with the previous retroviruses, these regions play an important role for viral budding since it was found that they interact with the Nedd4 and TSG101 proteins since an overexpression of Nedd4 alters the release of viral particles [81]. This feature of late domains is not restricted to retroviruses, as has been observed in other viruses, such as filoviruses like the Ebola virus, which do not have a Gag protein like retroviruses. It was identified that in the VP40 protein that plays a role in virus budding, it contains two overlapping late domains, which are PTAPPEY, that are both like the PTAP interactions with TSG101 and ESCRT, while PPEY interacts with Nedd4, but in contrast to that seen with retroviruses, it is not essential for budding [82]. In the rabies virus of the rhabdovirus family, something similar to the Ebola virus was found by identifying two overlapping late domains, PPEYVPL belonging to the PPEY domain and YVPL in the M protein of this virus, which is believed to interact with TSG101, ESCRT-I, and YVPL-recruited ALIX and ESCRT-III complex proteins such as CHMP2A and CHMP4B [83]. The arenaviruses were found, and the Z proteins have located PTAP and PPXY domains that interact with TSG101, and being modified these domains and the same protein TSG101 causes a significant decrease in viral production. Another important fact with arenaviruses is that most of these have been found a late domain, in the original arenaviruses, as mentioned, the Lassa virus contains two late domains PTAP and PPXY, while the Lujo virus only shows PSAP. Modern viruses such as Junin, Machupo, Guanarito, Sabia, and Chapare contain the PT/SAP domain and in this same context of viruses, it was found that the White Water Arroyo virus (WWAV) and Pichinde virus contain overlapping domains of PT/SAP and motifs similar to PPPY, with PT/SAPPY similar to those seen with Ebola [84,85]. The Hepatitis A virus was recently found to contain in its VP2 protein the YPX_n_L domain, and replacing Leu with Ala in the late domain eliminates virus release but does not alter viral assembly. They also observed a loss of ALIX recruitment, which helps interact with proteins of the ESCRT complex, and viral particles, can be released [86].

Another group of viruses in which these late domains have been found are flaviviruses. In the West Nile virus, it was analyzed that in the envelope protein, there are two conserved late domains, PXAP and YCYL, and when carrying out mutations of these domains occurs, a similar phenomenon to retroviruses happens when these changes occur, which is a drop in the production of viral particles. Although that was not similar to the previous examples when employing siRNA for ALIX and TSG101, as no effect on the release of viral particles occurred [87]. This opens the possibility that these domains interact with other proteins that interact with the ESCRT complex or have another function in the cellular environment. In a different case of the yellow fever virus (YFV) where it was found that in the NS3 protein, located the YPXI domain, which is similar to YPXL, and that in another work with *Aspergillus nidulans* the YPXL/I domain recognizes PalA/PacC, which is a homolog of AIP1/ALIX, there is a late motif similar to that reported in retroviruses and it shows that YPXI interacts with ALIX and that truncating this protein inhibits the release of viral particles [88,89]. On the other hand, in tick-borne encephalitis virus (TBEV), which refers to another flavivirus, it was evidenced that the NS3 protein contains a variation of the late domain seen in retroviruses, which is LYTLA, similar to the ΦYXΦXL sequence (Φ any non-polar amino acid); and that this late domain is involved in the recruitment of the ESCRT complex due to its interaction with ALIX/CHMP4A and that these events are related to viral replication and assembly [90,91]. Although this evidence for late domains is not directly related to issues of exosome sorting, it is related to the way in which components of ESCRT, ALIX, and TSG101 are recruited to the budding of these viruses. The interaction with cellular factors of the cellular protein sorting machinery and exosome biogenesis leaves an opening for further study of whether viruses could transport viral factors via this late domain signaling and recruit these factors into exosomes, as there is evidence that cellular factors containing late domains aid in their transport by exosomes.

Late domains have not only been found and evidenced in viral proteins. Since the evidence was found that heparan syndecan sulfate proteoglycans and their cytoplasmic adaptor syntenin regulate the formation of exosomes, and syntenin contains a YPX_n_L domain similar to that observed in retroviruses, this domain interacts with ALIX involved with the sorting and biogenesis of exosomes [92]. Another cellular protein evidenced to contain some late domain is the beta-galactoside-binding lectin galectin-3 (Gal-3), this protein is found intercellularly and extracellularly. The secretion of Gal-3 is measured independently of a secretory pathway by a non-classical undefined mechanism; however, it was found by electron microscopy that this protein was in the light of the exosomes and its release depends on the ESCRT-I complex, TSG101, and VPS4 ATPase. The most amazing thing about this interaction in this exosomal environment is that it was found that Gal-3 contains a PT/SAP motif conserved at the amino end, and when a mutation is made in this domain, a significant decrease in Gal-3 is seen [93]. Further evidence that these domains play an important role in exosomal sorting is seen for the Nedd4 family interacting protein 1 (Ndfip1) containing three PPXY domains. It was found that the expression of Ndfip1 acts as a switch for the packaging of a target protein, in this case, WW-tagged Cre recombinase as ubiquitination occurs and promotes the loading of CreWW into exosomes, evidencing the property of these late domains to load or sort proteins into exosomes [94]. The ability of late domains to promote the sorting of cellular proteins within exosomes and the potential study of viral proteins with these domains open a scenario for further investigation of the existence of these late domains in viral proteins and their implications.

## 8. Conclusions

The evidence described in this review show that an important number of viruses have conserved sequences called late domains in their structural and non-structural proteins. Although for HIV-1, it is important for the virus to migrate to the extracellular environment, in other cases, as in flavivirus another option exists, such as interacting with proteins that play roles in biogenesis and cargo selection of exosomes, as this could be the bridge or the key point for viral factors and the viruses themselves to reach the exosome. It is important to emphasize that the progress in developing methods for the isolation and characterization of EVs and exosomes is crucial for advancing the investigation of these cellular structures. The biggest problem in this field has been obtaining a high degree of purity of the exosomes. Implementing technologies such as ultracentrifugation, flow cytometry, and electron microscopy have been tools that can serve as a primary purification and characterization system. However, the use of size exclusion chromatography or asymmetric-flow field-flow fraction, which allows the separation of nanoparticles by their density and hydrodynamic properties, has allowed the identification of populations of exosomes with highly diverse molecular properties [95,96,97]. This new panorama opens a new study to identify these late domains in other unreported viruses and to understand their relationship with the transport of viral factors inside exosomes. Also, identifying the presence of post-translational modifications within these domains or in the viral cargo proteins will be a crucial piece of future research. Finally, the late domains PT/SAP, PPXY, and YXXL/YPXnL are required to understand their role in interacting with proteins such as ALIX, TGS101, and the ESCRT complex to determine their influence on the dissemination and replication of various viruses.

## Data Availability

Not applicable.
